# Putative invasive pulmonary aspergillosis within medical wards and intensive care units: a 4-year retrospective, observational, single-centre study

**DOI:** 10.1007/s11739-021-02705-z

**Published:** 2021-03-22

**Authors:** Silvia Corcione, Tommaso Lupia, Stefania Raviolo, Giorgia Montrucchio, Alice Trentalange, Antonio Curtoni, Rossana Cavallo, Francesco Giuseppe De Rosa

**Affiliations:** 1 Department of Medical Sciences, Infectious Diseases, University of Turin, Turin, Italy; 2 Tufts University School of Medicine, Boston, USA; 3 Microbiology and Virology Unit, University of Turin, Turin, Italy

**Keywords:** Aspergillosis, Pneumonia, Putative, Medical wards, Fungi

## Abstract

Blot and colleagues have proposed putative invasive pulmonary aspergillosis (PIPA) definitions for troublesome diagnosis in suspected patients outside the classical criteria of immunosuppression. We retrospectively included in the study all admitted patients with an *Aspergillus* spp. positive culture within lower airway samples. Overall, Aspergillus spp. positivity in respiratory samples was 0.97 every 1000 hospital admissions (HA): 4.94 and 0.28/1000/HA, respectively, in intensive care units (ICUs) and medical wards (MW). 66.6% fulfilled PIPA criteria, and 33.4% were defined as colonized. 69.2% of PIPA diagnosis occurred in the ICU. Antifungal therapy was appropriate in 88.5% of subjects with PIPA and 37.5% of colonized, confirming the comparison between deads and lives. Patients with PIPA in the ICUs had more frequent COPD, sepsis or septic shock, acute kidney injury (AKI), needed more surgery, mechanical ventilation (MV), vasopressors, hemodialysis, blood or platelets transfusions. PIPA in MW had associated with a history of smoking, interstitial lung disease and inhaled steroid therapy. Overall mortality within 21 days was 50%: 54.2% in ICU, 36,8% in MW. Factors associated with death were length of hospitalization, influenza, pneumonia, liver transplant, AKI, ARDS, sepsis and septic shock. PIPA in the ICU had higher disease severity and needed more organ support than MW cases, despite that cases of PIPA in MW are emerging with trends difficult to demonstrate given the problematic diagnosis.

## Introduction

Invasive fungal infections (IFIs) are well categorized in hematological and severely immunosuppressed patients. According to the recently revised and updated definitions of the European Organization for Research and Treatment of Cancer/Mycosis Study Group (EORTC/MSG) [1], IFIs are classified as proven, probable or possible. Diagnosis of IFIs is based on the presence of a combination of classical host factors (e.g., neutropenia, hematopoietic stem cell transplant [HSCT], immunosuppressants or prolonged corticosteroid therapy) or recently added factors (e.g., severe inherited immunodeficiencies and low CD4 count), clinical features and positive mycology [1]. In addition to these factors, low doses of corticosteroids, chronic obstructive pulmonary disease (COPD), liver cirrhosis, systemic connective tissue diseases, influenza infection, diabetes mellitus and advanced solid cancer have been described as a favorable environment for IFIs, especially for invasive pulmonary aspergillosis (IPA) [2, 3]. These extends this conditions beyond hematological departments to primarily involve patients admitted to the intensive care unit (ICU) and medical wards (MWs) [2, 3]. The signs and symptoms of IFIs have low specificity in these settings, and most diagnostic tests have only been validated in neutropenic hematologic or severely immunocompromised patients [3, 4]. Thus, diagnosis is often troublesome and delayed [3, 4]. To solve this problem, a new definition of putative IPA (PIPA) has been proposed by Blot and colleagues [5] for patients who do not fulfill the EORTC/MSG criteria [1]. The definition is based on the contemporary presence of clinical and radiological criteria with risk factors in patients who have positive *Aspergillus* spp. cultures [3, 5]. This study aimed to describe the epidemiology, risk factors, and clinical and prognostic features associated with a diagnosis of PIPA in a 4-year retrospective single-center analysis of hospitalized patients in the ICU. Additionally, the study extended the analysis to MW settings.

## Methods

A retrospective, observational, monocentric study was conducted. The study included all patients with a positive *Aspergillus* spp. culture in lower airway samples obtained through bronchoalveolar lavage (BAL) or broncho-aspirate (BA) who were admitted between 01 January, 2012 and 31 December, 2016 to the MWs and ICU at Città Della Salute e Della Scienza di Torino–Presidio Molinette. Patients with a previous history of onco-hematological diseases (iron deficiency anemia, hemophilia, sickle cell disease, thalassemia, leukemias and lymphomas) or a history of IPA were excluded. Each patient’s demographics and clinical and microbiological data were collected from electronic medical records and expressed as medians with interquartile ranges (IQRs). The incidence of positive culture was calculated on the thousandth hospital admission (HA). Signs and symptoms compatible with the clinical algorithm by Blot et al. [5] were reported along with the microbiological data. Positive *Aspergillus* spp. culture, blood galactomannan (GM) test, GM obtained through BAL or BA samples and a direct microscopic exam or polymerase chain reaction (PCR) for *Aspergillus* spp test positivity were collected. Mortality was evaluated 21 days after direct microbiological diagnosis (e.g., positive *Aspergillus* spp. culture, direct microscopic exam) or indirect diagnosis (e.g., GM). The study population was divided into three groups according to Blot et al. [5]. The first group included patients with a proven diagnosis of IPA according to the EORTC/MSG [1] definition (i.e., *Aspergillus* spp. positivity on microscopic analysis or cultures). The second group included patients with a diagnosis of PIPA who met all four following criteria:

1. *Aspergillus*-positive lower respiratory tract specimen culture (entry criterion).
2. Compatible signs and symptoms, including at least one of the following:
  - Fever refractory to at least 3 days of appropriate antibiotic therapy.
  - Recrudescent fever after a period of defervescence of at least 48 h, while still on antibiotics and without other apparent cause.
  - Pleuritic chest pain.
  - Pleuritic rub.
  - Dyspnoea.
  - Haemoptysis.
  - Worsening respiratory insufficiency despite appropriate antibiotic therapy and ventilatory support.
3. Abnormal medical imaging by portable chest X-ray or CT scan of the lungs.
4. Either IVa or IVb.
  - IVa host risk factors (one of the following conditions): neutropenia (absolute neutrophil count, 500/mm3) preceding or at the time of ICU admission, underlying hematological or oncological malignancy treated with cytotoxic agents, glucocorticoid treatment (prednisone equivalent, 20 mg/day) and congenital or acquired immunodeficiency.
  - IVb semiquantitative *Aspergillus*-positive culture of BAL fluid (+ or ++) without bacterial growth together with a positive cytological smear showing branching hyphae

Group three included patients with *Aspergillus* spp. respiratory tract colonization (when ≥ 1 criterion necessary for a diagnosis of PIPA was not met). Appropriate antifungal therapy at the time of microbiological positivity was retrospectively evaluated according to the currently available international guidelines from the Infectious Diseases Society of America (IDSA) and European Society of Clinical Microbiology and Infectious Diseases (ESCMID). Data were collected in an Excel spreadsheet and analysed the Statistical Package for the Social Sciences (SPSS) 18.0. continuous variables were reported as mean (standard deviation) or median (IQR). categorical variables were reported as absolute numbers (percentages). Non-parametric tests (Wilcoxon, Mann–Whitney, Chi-square and Fisher’s exact tests) were used for univariate analyses. For categorical variables, Chi-square and Fisher’s tests were used depending on the distribution of the contingency tables. Non-parametric tests (Wilcoxon and Mann–Whitney) were used for continuous variables, and Chi-square and Fisher’s tests were used for categorical variables. The significance level was set at 0.05 to judge the strength of evidence for hypothesis testing. Factors presenting a significant level in the univariate analyses were included in the multivariate analyses (log regression) to assess for risk factors of death outcome.

## Results

During the study period, 80,570 patients were admitted to the ICUs and MWs. Overall*, Aspergillus* spp. positivity in the lower airway cultures (e.g., BAL or BA) was 0.97 for every 1000 HA distributed as follows: 4.94/1000 in the ICUs and 0.28/1000 in the MWs. Of these patients, 76 (0.09%) fulfilled the criteria to be included in the study. The median age was 66.5 years old (range 58–77 years; 65.4%, ≥ 60 years), and the patients were mostly men (75.6% male vs. 24.4% female). The median hospital length of stay (LOS) was 32 days (range 17.75.59 days), and the patients were hospitalized more frequently in the ICUs (76.3%) than in the MWs (23.7%). The algorithm for PIPA by Blot et al. [5] was applied to the patients. Of the 76 individuals, 52 (68.4%) had a diagnosis of PIPA. Of these patients diagnosed with PIPA, 69.2% were hospitalized in the ICU, and 30.8% were hospitalized in the MWs. The remaining 24 patients did not fulfill the PIPA criteria, so microbiological positivity was considered as lower respiratory tract colonization in these patients. The main characteristics of the entire population are shown in Table 1. Regarding comorbidities, the majority of patients had cardiovascular disease (73.1%), acute kidney injury (51.3%) or diabetes (42.3%). Other relevant comorbidities were COPD (42.3%) or a history of malignancy (34.6%); within the first group, 51.5% of the patients were undergoing daily chronic steroid inhalation therapy at home. At the time of microbiological positivity, 83.3% needed O2 therapy, 73.1% needed mechanical ventilation support, 64.1% needed hemodynamic and vasopressor support, 28.1% needed hemodialysis, and 96.2% had concomitant antibiotic therapy. Serum GM was positive in 20.9% of patients, with a median GM value of 1.96 ng/mL (0.775–4.55 ng/mL, normal value < 0.5 ng/mL). However, a GM BAL/BA test was not performed in 17.8% of adults. GM on BAL or BA was positive in 98.2% of the tests, with a median value of 5.05 ng/mL (4.07–6.4 ng/mL; normal value < 0.5 ng/mL). In the cultures, *A. fumigatus* was the species most frequently isolated (72.9%), followed by *A. terreus*, *A. flavus* and *A. niger*. Additionally, 7 (8.9%) adults had double species isolation during hospitalization. Furthermore, *Aspergillus* spp. isolation had some seasonal fluctuations and was found more often in winter (46.2%) and spring (21.8%). Of the patients, 73.1% were treated with an appropriate antifungal therapy at the moment of microbiological positivity. The majority (89.5%) were treated with amphotericin B or voriconazole monotherapy (42.1% vs. 36.8%, respectively), while the remaining patients were treated with caspofungin or posaconazole monotherapy or combination therapy. A univariate analysis was performed to assess the differences between patients with a diagnosis of PIPA and those colonized by *Aspergillus* spp. in both the ICUs and MWs. In patients from the ICUs, a diagnosis of PIPA was associated with more oral or intravenous steroid therapy during hospitalization, history of metastatic solid organ cancer, sepsis, features of pneumonia on X-rays or radiological abnormalities (Table 1). Furthermore, patients with PIPA in the ICUs were more frequently treated with antifungal therapy compared to colonized patients (*p* < 0.0001) and were treated before microbiologic results more often (*p* = 0.03). Additionally, ICU-infected patients underwent hemodialysis more commonly than colonized patients and had more respiratory insufficiency and worsening of respiratory insufficiency despite antibiotic therapy and adequate mechanical ventilation (Table 1). Subsequently, a univariate analysis (Table 1) was performed to assess the differences between patients with PIPA in the ICUs and MWs. Patients with PIPA in the ICUs presented more frequently with COPD, sepsis, septic shock and acute kidney failure. Moreover, ICU subjects needed surgical intervention or support therapies, such as mechanical ventilation, vasopressors, hemodialysis and blood or platelet transfusions, more often than patients in the MWs. Patients in the MWs were more likely to be smokers, had interstitial lung disease and had undergone chronic inhaled steroid therapy (Table 1). The estimation of mortality at day 21 was assessed from the microbiological positivity, and the overall mortality within 21 days was 51.3% (39 subjects). The mortality in the ICU was 82%, as opposed to 17.9% in MW patients (*p* = 0.622). 37 patients (49.7%) survived: 25.3% went back home, 7.2% were transferred to another hospital, and 16.2% went to other facilities. Antifungal treatment was administered to 81.5% of the non-survivor patients diagnosed with PIPA and in 27.3% of the colonized dead patients. Characteristics in the univariate analysis (Table 2) associated with not survival were as follows: duration of hospitalization (longer in the live population, *p* = 0.006), influenza diagnosis (*p* = 0.048), possible pneumonia on radiological exams (*p* = 0.042), liver transplant (*p* = 0.048), acute kidney failure (*p* < 0.0001), acute respiratory distress syndrome (ARDS) (*p* = 0.019), sepsis (*p* < 0.0001), septic shock (*p* < 0.0001) and worsening of respiratory insufficiency in spite of mechanical ventilation (*p* = 0.003). Immunosuppressive drugs were associated with a lower risk of death (*p* = 0.002). According to the Blot et al. criteria, antifungal therapy was appropriate in 88.5% of patients with a diagnosis of PIPA and 37.5% of colonized patients, with a statistically significant difference in the univariate analysis (*p* < 0.0001). This association was confirmed after comparing non-survivor patients with alive ones (*p* = 0.001). Multivariate regression analysis (Tables 3, 4) was performed for patients hospitalized in the ICU. The factors associated with PIPA included steroid therapy (regardless of dosage) and absence of antifungal therapy (Table 3). Multivariate regression analysis for prognostic mortality factors showed (Table 4) that acute kidney failure (OR: 9.139; confidence interval, CI 2.892–28.878; *p* = 0.0001) and worsening of respiratory insufficiency (OR: 4.159; CI 1.153–15.000; *p* = 0.029) despite mechanical ventilation and antibiotic therapy were tied with higher mortality. In contrast, liver transplants had a protective effect (OR: 0.066 CI 0.005–0.812; *p* = 0.034].

**Table 1:** Main characteristics of the study population

	ICU (*n* = 58)		*p* value	MW (*n* = 18)		*p* value	*p* value ICU vs. MW
	PIPA (*n* = 36)	Colonization (*n* = 22)		PIPA (*n* = 16)	Colonization (*n* = 2)		
	*n* median (IRQ)	*n* median (IRQ)		*n* median (IRQ)	*n* median (IRQ)		
Demographic variables							
Gender (male)	28	17	0.964	12	2	1	1
Age	66.5 (58–75.75)	71 (58–79.25)	0.475	64.5 (59.25–74.25)	78 (77–78)	0.209	0.945
LOS	37.5 (19.5–68.5)	32 (17.75–53.5)	0.553	25 (14.5–42.25)	41.5 (28–41.5)	0.399	0.730
LOS from microbiological diagnosis	11.5 (6–21.75)	6 (1.75–21.5)	0.328	10.5 (3.25–17)	16.5 (13–16.5)	0.327	0.493
Comorbidities and risk factors							
Previous antibiotic treatment	32	16	0.156	15	2	1	1
Cardiovascular diseases	28	15	0.418	12	2	0.490	1
Pneumonia	19	4	0.013	12	2	1	0.220
COPD	12	10	0.356	11	0	0.137	0.018
ILD	3	4	0.409	5	0	1	0.035
Pancreatitis	4	2	1	1	0	1	1
AKI	25	10	0.070	5	0	1	0.010
CKD	11	3	0.209	5	2	1	0.960
DM	12	9	0.560	5	2	0.137	0.882
Cirrhosis	6	4	1	4	1	0.490	0.475
Smoke							
Present	4	7	0.069	3	0	0.171	0.662
Former	7	1		8	0		0.025
SOT							
Lung	0	1	0.379	5	0	1	0.002
Kidney	3	1	1	1	0	1	1
Liver	5	2	0.698	0	0	–	0.308
Malignancy							
Primitive	6	8	0.089	0	1	0.111	0.083
Metastatic	9	0	0.010	3	0	1	0.733
Influenza co-infection	4	2	1	1	0	1	1
Steroids > 60 days (> 1 mg/kg/d)	4	1	0.387	5	0	1	0.113
Steroids > 21 days (0.3 mg/kg/d)	7	1	0.139	7	0	0.497	0.094
Steroids during hospitalization	28	4	0.000	13	0	0.235	1
Chronic steroid inhaler	7	4	1	9	0	0.471	0.008
CT for malignancy	5	0	0.145	4	0	1	0.431
Neutropenia (< 500 cell/mm^3^)	2	1	1	0	0	–	1
Immunosuppressive treatments	7	3	0.727	6	0	0.529	0.184
HIV or inherited immunodeficiency	2	0	0.521	0	0	–	1
ARDS	10	3	0.332	1	0	1	0.140
Sepsis	30	13	0.041	5	1	1	0.000
Septic shock	27	11	0.052	1	1	0.216	0.000
Prior TB	1	0	1	1	0	1	0.525
No major risk factors	24	18	0.243	7	2	0.471	0.120
Therapies							
Antifungal	30	7	0.000	16	2	–	0.160
Switch (from antifungal to prophylaxis)	6	0	0.569	7	1	1	0.076
Prophylaxis	21	6	0.030	6	2	0.183	0.165
Symptoms							
Dyspnoea	14	6	0.366	8	2	0.477	0.454
Fever	12	2	0.057	4	1	0.490	0.747
Recurrent fever	9	4	0.747	5	0	1	0.639
Pleurodynia	1	0	1	1	0	1	0.525
Pleuric rub	6	3	1	3	1	0.405	1
Hemopptysis	0	1	0.379	2	0	1	0.090
Respiratory failure	18	4	0.025	1	0	1	0.004
Imaging	36	13	0.000	16	2	–	–
Typical for IPA	11	0	0.275	4	1	0.593	0.890
Support therapies							
Oxygen	31	21	0.392	11	1	1	0.143
Mechanical ventilation	34	21	1	1	0	1	0.000
Vasopressors	30	16	0.333	3	0	1	0.000
Hemodyalisis	16	3	0.021	2	0	1	0.031
Blood transfusion	33	17	0.238	5	0	1	0.000
Platelets transfusion	9	8	0.356	0	0	–	0.043
Surgery	28	12	0.063	1	0	1	0.000
Antibiotics	35	21	1	15	2	1	0.525
Microbiology							
GM (serum)	3/22	0/7	0.557	4/11	0/1	1	0.186
GM BAL/BA	32/32	6/6	–	13/13	2/2	–	–
Aspergillus DNA	12/18	2/4	0.602	8/9	2/0	1	0.363

*LOS* lenght of stay, *GM* galattomannan, *ARDS* acute respiratory distress syndrome, *BAL* bronchoalveolar lavage, *BA* broncho-aspirate, *ICU* intensive care unit, *MW* medical ward, *COPD* chronic obstructive pulmonary disease, *ILD* interstitial lung disease, *DM* diabete mellitus, *CKD* chronic kidney disease, *AKI* acute kidney injury, *SOT* solid organ transplant

**Table 2:** Characteristics of the study population in univariate analysis

	Survivors (*n* = 39) *n* median (IRQ)	Deceased (*n* = 39) *n* median (IRQ)	*p* value
Demographics			
Males	28	31	0.429
Age	63 (57–72)	71 (59–78)	0.066
LOS	44 (18–70)	28 (16–37)	0.006
LOS from microbiological diagnosis	9 (2–17)	13(6–13)	0.107
ICU	27	32	0.622
MW	12	7	
Comorbidities			
Previous antibiotics	32	35	0.329
Cardiovascular diseases	31	26	0.202
Pneumonia	14	24	0.042
COPD	17	16	0.819
ILD	5	7	0.530
Pancreatitis	4	3	1
AKI	11	30	0.000
CKD	8	13	0.202
DM	14	14	1
Cirrhosis	9	6	0.389
Smoke			
Actual	6	8	0.765
Former	9	7	
SOT			
Lung	5	1	0.200
Kidney	5	0	0.055
Liver	6	1	0.048
Malignancy			
Solid	7	8	0.774
Metastatic	8	4	0.347
Influenza co-infection	1	6	0.048
Steroids > 60 days (> 1 mg/kg/day prednisone)	6	4	0.737
Steroids > 21 days (0.3 mg/kg/die prednisone)	8	8	1
Steroids	23	23	0.890
Chronica inhaled steroids	12	8	0.300
Malignancy during chemotherapy	6	3	0.288
Neutropenia (< 500 cell/mm^3^)	2	1	1
Immunosuppressive therapies	14	2	0.001
HIV/AIDS	1	1	1
ARDS	3	12	0.019
Sepsis	14	36	0.000
Septic shock	8	33	0.000
Previous lung tubercolosis	2	1	1
No major risk factors	22	30	0.055
Therapy			
Antifungal	31	26	0.202
Switch	10	4	0.139
Antifungal prophylaxis	16	19	0.495
Symptoms			
Dispnoea	12	19	0.105
Fever	8	12	0.300
Recurrent fever	8	11	0.429
Pleurodynia	1	1	1
Pleuric rub	5	8	0.362
Hemottysis	3	1	0.615
Acute respiratory failure	6	18	0.003
Imaging	33	36	0.288
Typical	7	10	0.849
Supportive therapies			
Oxygen	29	36	0.0.65
Mechanical ventilation	24	33	0.040
Vasopressors	17	33	0.000
Hemodialysis	6	16	0.012
Blood transfusion	23	32	0.025
Platelets transfusion	5	13	0.032
Surgery	22	20	0.650
Antibiotics	37	38	0.556
Microbiology			
GM serum	4	5	0.721
GM BAL/BA	26	28	0.491
Aspergillus DNA	14	10	0.146

*LOS* lenght of stay, *GM* galattomannan, *ARDS* acute respiratory distress syndrome, *BAL* bronchoalveolar lavage, *BA* broncho-aspirate, *ICU* intensive care unit, *MW* medical ward, *COPD* chronic obstructive pulmonary disease, *ILD* interstitial lung disease, *DM* diabete mellitus, *CKD* chronic kidney disease, *AKI* acute kidney injury, *SOT* solid organ transplant

**Table 3:** Risk factors for putative invasive pulmonary aspergillosis in multivariate analysis

	*p* value	a OR	95% CI	
			Inferior	Superior
Steroids	0.001	12.950	2.990	56.086
Worsening of respiratory failure	0.342	2.310	0.411	12.989
Sepsis	0.419	2.014	0.368	11.013
Lack of antifungal therapy	0.004	10.430	2.123	51.256
Hemodialysis	0.374	0.414	0.059	2.888

*aOR *adjusted odd ratio,* CI* confidence interval

**Table 4:** Characteristics associated with 21-day mortality in multivariate analysis

	*p* value	a OR	95% CI	
			Inferior	Superior
Acute kidney failure	0.000	9.139	2.892	28.878
Worsening of respiratory failure	0.029	4.159	1.153	15.000
Liver transplant	0.034	0.066	0.005	0.812
Influenza co-infection	0.206	4.751	0.425	53.160

*aOR* adjusted Odd ratio, *CI* interval of confidence

## Discussion

Recently, IPA has been described in apparently immunocompetent patients, and thus a new at-risk group has been defined, notably outside hematological wards [2–5]. However, definite diagnosis is problematic in every population at risk of IPA, because it requires a lung biopsy demonstrating invasive growth and positive culture, which is often hazardous due to the underlying conditions of the patients. Additionally, the diagnosis of probable or possible IPA according to EORTC/MSG [1] requires an immunocompromised host, which excludes the vast majority of the ICU and MW population [2–5]. This study reported a noticeable incidence of *Aspergillus* spp. positivity on lower airway cultures in the ICU population (4.94/1000 HA) over 4 years. The incidence is particularly noticeable when compared to longer studies in the same setting, such as the 14-year French retrospective study by Lugosi et al. [6]. They examined 11,992 patients admitted to ICUs and found that 9.08/1000 HA had *Aspergillus* spp. positivity in the lower airway cultures. Moreover, in an 18-month surveillance program involving 18 Italian ICUs, the incidence of IPA was 2.3/1000 HA [7], although IPA has been considered a rare condition among critically ill patients, but with a deadly mortality between 60 and 90% [8, 9]. Furthermore, data on overall positivity in MWs are substantially lacking in the literature. Russo et al. [10] reported clinical features from 1351 subjects extrapolated during a 14-month period of infectious disease consultation in a tertiary care hospital in Italy. They found 14 cases of proven or probable invasive aspergillosis enrolled in medical and surgical wards. In the present analysis, the incidence of lower respiratory tract positivity for *Aspergillus* spp. among MWs was reported as 0.28/1000 HA. Within this group, 16 patients fulfilled the criteria for PIPA by Blot et al. [5]. Contrary to the case study by Russo et al. [10] that observed about 70% of PIPA cases in non-ICU wards, the present study described PIPA in just 30.8% of MWs adults with MWs. This discrepancy may have been related to a higher immunosuppressed MW population in the mentioned study [10] (e.g., high rate of malignancies, frequent cancer chemotherapies) and different diagnostic criteria. Advanced age and the predominance of males in the present study were comparable to the literature in this field [2, 5, 6, 10]; this predominance of older men may be related to the higher incidence of comorbidities with PIPA in the aged population, notably chronic lung diseases, malignancies and chronic liver diseases. According to Matthaiou et al. [11], elderly patients were more likely to be diagnosed with PIPA than proven or possible IPA, due to less diagnostic radiological findings concurring with a delayed diagnosis. Cardiovascular diseases (73.1%) and diabetes (42.3%) were the two most frequent comorbidities in this population, and the same distribution of underlying diseases (13.0% and 7.8%, respectively) was found in the study by Blot et al. [5], although at lower rates. A high prevalence of chronic lung diseases (42.3%) was found in this study, and this disease was more frequent among patients with PIPA in the MWs. Indeed, acute exacerbations in COPD were a cause of frequent admission to the MWs and ICUs. In an observational multicenter study of 563 critically ill patients with PIPA or *Aspergillus* spp. colonization, COPD was the most frequent underlying condition, involving 31% of adults [12]. Delsuc [13] and colleagues have described in a cohort of 677 COPD subjects admitted to the ICU that PIPA was a strong death predictor in critically ill patients; therefore, the use of corticosteroids and antibiotics before ICU admission was a risk factor for the development of PIPA. Moreover, acute kidney injury (AKI) and chronic kidney disease are underreported in most other studies. The present study found that AKI resulted in a mortality risk factor. Nevertheless, higher organ support was associated with more PIPA diagnoses, notably in the ICU setting, than reported by other authors [5–9]. In the present study, MW patients included more past or former smokers, interstitial lung diseases and chronic inhaled steroid therapies than ICU patients. Regarding the association between smoking and IFIs, Pourbaix et al. [10, 14] found an increased risk of fungal infections among smokers and recommended the implementation of smoking cessation strategies, especially in high-risk groups, such as HIV or hematological patients. However, ICU and MW patients were not included in their discussion. *A. fumigatus* was the most isolated species, followed by A. *terreus*. *A. terreus* has not been discussed frequently in the literature, except in some specific geographic areas [11, 15, 16]. This is of particular importance for therapy choice since *A. terreus* is resistant to amphotericin B, a first-choice therapy in invasive aspergillosis, along with voriconazole and isavuconazole [11, 15, 16]. The mortality of the current study’s population was similar to neutropenic and non-neutropenic populations affected by aspergillosis. No difference in mortality was found between patients with PIPA and colonized patients. This observation was recently confirmed by Paiva et al. among ICUs in which patients with putative/proven IPA were more likely to receive antifungal therapies than colonized patients (78.7% vs. 25.5).. As shown in the present study, *Aspergillus* isolation must not always be considered as simple colonization or contamination in immunocompromised patients, as IPA can also occur in patients who lack classical risk factors, like neutropenia or therapies with immunosuppressant drugs. The mortality for IPA in non-neutropenic is comparable to neutropenic patients, and high comorbidities in the MWs, along with the ICU population as reported in other studies [17, 18] and must be screened in the absence of other compatible diagnoses for IPA.

There are several limitations to this study. First, this is a single-centre study. Our patient population may not accurately reflect the general demographics of Italy. Second, there are some missing data, such as information regarding APS or APACHE score for ICU patients and in mortality analysis and GOLD classification in COPD patients. Third, the low numerosity of the sample included in our study.

In conclusion, in our study, MW patients primarily suffered from underlying respiratory diseases such as pneumonia and COPD, whereas ICU patients had a wider range of comorbid conditions, higher disease severity and needed more organ support.

## Data Availability

Available on affordable request.
